# Movement and contact patterns of long-distance free-grazing ducks and avian influenza persistence in Vietnam

**DOI:** 10.1371/journal.pone.0178241

**Published:** 2017-06-20

**Authors:** Anne Meyer, Tung Xuan Dinh, Thu Van Nhu, Long Thanh Pham, Scott Newman, Thuy Thi Thanh Nguyen, Dirk Udo Pfeiffer, Timothée Vergne

**Affiliations:** 1 Veterinary Epidemiology, Economics and Public Health Group, Royal Veterinary College, London, United-Kingdom; 2 National Institute for Animal Sciences, Hanoi, Vietnam; 3 Department of Animal Health, Ministry of Agriculture and Rural Development, Hanoi, Vietnam; 4 Emergency Centre for Transboundary Animal Diseases, Food and Agriculture Organization of the United Nations, Hanoi, Vietnam; 5 School of Veterinary Medicine, City University of Hong Kong, Kowloon, Hong Kong SAR, China; 6 MIVEGEC (Maladies Infectieuses et Vecteurs: Ecologie, Génétique, Evolution et Contrôle) Group, Institut de Recherche pour le Développement (IRD-224, CNRS-5290, Université de Montpellier 2), Montpellier, France; South China Agricultural University, CHINA

## Abstract

Presence of ducks, and in particular of free-grazing ducks, has consistently been shown to be one of the most important risk factors for highly pathogenic avian influenza outbreaks which has compromised poultry production in South-East Asia since the early 2000s and continues to threaten public health, farmers’ livelihood and food security. Although free-grazing duck production has been practised for decades in South-East Asia, there are few published studies describing this production system, which is suspected to play an important role in the maintenance of avian influenza viruses. This study aimed at describing quantitatively the long-distance free-grazing duck production system in South Vietnam, characterising the movement and contact patterns of the duck flocks, and identifying potential associations between farming practices, movement and contact patterns and the circulation of avian influenza viruses. We conducted interviews among stakeholders involved in the free-grazing duck production system (duck farmers, transporters and rice paddy owners) in combination with a virological cross-sectional survey in South Vietnam. Results show that both direct and indirect contacts between free-grazing duck flocks were frequent and diverse. The flocks were transported extensively across district and province boundaries, mainly by boat but also by truck or on foot. A third of the investigated flocks had a positive influenza A virology test, indicating current circulation of avian influenza viruses, but none were positive for H5 subtypes. The age and size of the flock as well as its location at the time of sampling were associated with the risk of influenza A circulation in the flocks. These findings should be considered when developing risk assessment models of influenza virus spread aimed at informing the development of improved biosecurity practices leading to enhanced animal health, sustainable animal production and reliable income for farmers.

## Introduction

For over a decade, regular outbreaks of highly pathogenic avian influenza (HPAI) have occurred in poultry throughout South-East Asia, in spite of large-scale vaccination campaigns, as implemented in Vietnam and Indonesia, and stamping-out interventions [1, 2]. In Vietnam, 43 HPAI outbreaks were reported in 2015, distributed across 23 of Vietnam’s 63 provinces [3]. A large number of studies have contributed to greatly improve our understanding of the epidemiology of HPAI viruses by highlighting the importance of several drivers of its distribution and spread. Noticeably, the presence of ducks was regularly shown to be strongly associated with the distribution of H5N1 outbreaks in Vietnam [4–6] and in the wider region [7–11]. This has been linked with the occurrence of both asymptomatic avian influenza virus infection [12, 13] and excretion [14] in domestic ducks. In addition, live bird markets have been shown to contribute to the spread of avian influenza viruses and facilitate their persistence [15, 16]. While formal live bird markets have been prohibited in the Mekong region, it is likely that there is continuing informal live bird trading activity, but it is recognised that this alone cannot explain the continuing circulation of avian influenza in South Vietnam.

In Vietnam, poultry production in general, and duck production in particular, is concentrated in the Red River delta and the Mekong River delta (MRD), with a density of ducks in the MRD of about 507 heads per km^2^ [17]. Free-grazing duck (FGD) farming is a common practice in these two regions, and about half of the duck production of the country originates from free-grazing management systems [17]. In such systems, adult ducks scavenge freely on recently-harvested rice paddies where they feed on leftover rice grains, insects and molluscs. Two types of FGD systems have been described in Vietnam [18]: *short-distance* (also called *stationary*) FGDs are herded within the commune boundaries and return to the farm premises at night, while *long-distance* (also called *moving*, or *transhumant*) FGDs leave the farm for an extended period of time, often several weeks, and are transported across administrative boundaries. According to a survey conducted in 2010 in the An Giang province of the MRD, long-distance FGDs, short-distance FGDs and confined ducks accounted for 55%, 43% and 2% of the number of ducks, respectively [19]. In Northern Vietnam, long-distance FGD farming is not a common practice [17].

It has recently been suggested that long-distance FGDs are likely to play an important role in the maintenance and spread of the avian influenza (AI) viruses [20]. First, free-grazing ducks may act as local reservoirs and amplification hosts of virus transmitted by migratory birds, followed by secondary spread to other domestic poultry [9]. Second, when released in the field for grazing or when being transported from one grazing place to another, long-distance FGDs may be in direct or indirect contact with other free-grazing duck flocks, potentially leading to virus transmission events across larger distances. Third, transport of FGD flocks across province and even national borders [21] may lead to the spread of influenza viruses over relatively long-distances. Finally, transport of live animals has often been identified as a risk factor for influenza outbreaks, due to a high density of animals kept and herded together resulting in increased stress levels which is likely to increase virus shedding [22]. By conducting biological sampling of duck flocks and questionnaire surveys amongst duck farmers, transporters and rice paddy owners, this study aimed at i) describing the farming practices and contact patterns of long-distance FGD flocks in South Vietnam, ii) estimating the level of circulation of influenza A viruses amongst long-distance FGD flocks and iii) identifying associations between farming practices, contact patterns and the circulation of avian influenza viruses.

## Methods

### Ethics statement

This study has been approved by the Clinical Research and Ethical Review Board of the Royal Veterinary College (project number 3597).

### Questionnaire study

The study was conducted in An Giang province, in South Vietnam. Two districts (Chau Phu and Tri Ton) were selected on the basis of the importance of the integrated rice-duck production system and the presence of FGD flocks at the time of the field work, which took place between October and December 2015. There are no official records of the location and movements of FGD flocks, so the only way to identify farmers to be interviewed was to use the most up-to-date local knowledge of communal veterinarians [23]. Using this convenience sampling approach, 44 long-distance FGD farmers were identified and interviewed. In addition, we interviewed 23 rice paddy owners and 17 FGD transporters who were identified based on the local knowledge of the commune veterinarians and of the FGD farmers already interviewed.

Three questionnaires, one for each type of stakeholder (long-distance FGD farmers, hereafter referred to as ‘farmers’, rice paddy owners and FGD transporters), were developed and tested in the field during a two-day pilot study (the questionnaires are available as supporting information files). The farmer questionnaire included questions related to the socio-economics of the farm, duck production, flock movements and poultry health. For the purpose of this study, a flock was defined as a group of birds of the same age purchased, managed and sold as a whole. Some farmers owned two or more groups of birds of different ages, which were kept separate during grazing, transport and in enclosures; these groups were defined as distinct flocks. Farmers were also asked to locate all sites that they had visited with their current flock. Subsequently, further aspects about each site (observed contacts between flocks, type of transport used to travel between sites) were investigated. The location of each site specified by the farmers was approximated by the coordinates of the centroid of the commune were the site was located. The distance between sites was calculated as the Euclidean distance between two sites visited consecutively by a farmer. The transporter questionnaire included questions related to the socio-economics of the household, the characteristics of the duck transport activity and the biosecurity practices. The paddy owner questionnaire included questions related to the socio-economics of the household, the characteristics of the paddy management in terms of rice culture and post-harvest renting as well as the observed contacts between FGD flocks. Informed consent was obtained in writing prior to each interview. The questions were asked in Vietnamese by a previously trained interpreter, and the answers were translated into English by the interpreter directly to the primary investigator (AM) who recorded the answers and asked for clarification when necessary.

### Detection of avian influenza viruses

Concurrently with the questionnaire study, biological samples were taken both from interviewed farmers’ duck flocks and interviewed transporters’ vehicles (boats or trucks). Oropharyngeal swabs were collected by the local veterinary services from 60 birds per flock and 20 samples of fresh faeces were taken from each transport vehicle. Oropharyngeal swabs were collected rather than cloacal swabs, as it was previously suggested that they are more sensitive to detect the presence of HPAI H5N1 virus in ducks [14, 24, 25]. The sample size for the number of ducks per flock was set such that it provided 95% confidence to detect at least one positive bird in an infected flock with a within-herd prevalence of 5%, a test sensitivity of 95% and a test specificity of 100%. Samples were refrigerated in cool boxes on wet ice and transported to the local district office of the Department for Animal Health where they were stored in a fridge at +4°C for a maximum of four days. The swab samples were then transferred to the regional veterinary diagnostic laboratory and stored at -80°C until further processing. Pools of five individual samples (from the same flock or vehicle) were extracted and tested by real-time Reverse Transcription Polymerase Chain Reaction (rRT-PCR) using a commercial kit (SuperScript III One Step, Invitrogen, Carlsbad, USA). In the screening phase, all pooled samples were tested for the presence of the matrix (M) gene of influenza A viruses, and M-positive pooled samples were subsequently tested for the presence of the H5 subtype of the HA gene. The primer/probe sets used for both rRT-PCR assays were adapted to the local context by the National Centre for Veterinary Diagnosis of Vietnam based on sequences previously published [26]. Samples with a threshold cycle value inferior or equal to 35 were considered positive.

### Statistical analyses

Questionnaire results were summarised using descriptive statistical indicators (proportion, mean, standard deviation and range) and differences between groups were tested using standard statistical tests appropriate for the type of variables (χ^2^ test and Fisher’s Exact test). A logistic regression analysis was performed to identify the variables that were associated with an increased risk of influenza A infection of the duck flocks. The outcome variable was the influenza A infection status of the flock. A flock was considered positive if at least one sample out of 60 tested positive for influenza A. Explanatory variables included a total of 51 farm-level variables derived from the data collected via the questionnaires. First, potential risk factors were screened for statistically significant association with influenza A infection status using univariable logistic regression based on the likelihood ratio test. Variables significant at p < 0.2 were retained for the multivariable analysis. They were manually added to a logistic regression model using a stepwise forward selection procedure. We used the Akaike Information Criterion (AIC) to select the model best fitting the data. The best model was considered to be the most parsimonious model whose AIC was less than two points greater than that of the model associated with the smallest AIC [27]. We used the variance inflation factor of the final model to detect possible multicollinearity between the predictors [28]. We considered that a variance inflation factor higher than 4 indicated at least moderate collinearity between the model variables.

During the field activities, the data was recorded on paper questionnaire forms and subsequently entered and checked by the primary investigator into a Microsoft Access database. Data analysis was performed using the R software version 3.3.0 [29] and the MASS package [30]. Spatial data analysis was performed using ArcGIS Desktop version 10.1 [31].

## Results

### Description of long-distance free-grazing duck flocks

All farmers interviewed during the course of the study kept layer ducks of a breed named “vịt cò” which refers to their relatively small size and adaptability to outdoor foraging. This breed was created by crossing local breeds with improved layer duck breeds (e.g., Super Egg and Khaki Campbell) in order to increase the egg production while keeping the scavenging capacity and disease resistance. All farmers kept ducks for egg production only. Thirty-one farmers (70%) sourced their flocks as day-old ducklings from a hatchery, while the remainder purchased adult ducks from another duck farmer or a trader (the median, minimum and maximum age of the ducks at the time of purchase were 150, 75 and 240 days, respectively). All duck flocks had been vaccinated against HPAI subtype H5N1 virus at least once, using either a vaccine produced by a Vietnamese company [32] or a vaccine produced in China [33]. Eight flocks (18%) had received only one HPAI vaccine injection during their lifetime (i.e. since the ducks hatched). Out of the 36 flocks for which the information was available, 34 (94%) had been vaccinated within the previous six months. The proportion of farmers recalling the last vaccination date was significantly higher amongst farmers buying their flocks from other farmers or traders (6/14) than for those who bought their ducks from hatcheries (2/28, Fisher’s Exact test, p-value < 0.05). All farmers but one also vaccinated their flocks against duck cholera. No other vaccines were reportedly used. The median flock size was 2,200 ducks (range: 300–8,000). Farmers reported selling their ducks either for further egg production or as spent layer ducks, depending on factors such as the price of eggs and the availability of rice paddies (median, minimum and maximum age at sale: 15, 8 and 25 months, respectively).

### Source of income of farmers, paddy owners and duck transporters

Each of the three stakeholder groups (farmers, paddy owners, and transporters) had specific sources of income, as presented in Table 1. Most of the interviewees in each group specialised in their respective activities: 87%, 91% and 76% of the farmers, paddy owners and transporters, respectively, named their activity as their main source of income. However, household income was diverse and individual households had a median of 3 different income-generating activities (range: 1 to 7). Additional income-generating activities most often cited by the interviewees were agriculture (rice, ducks, geese, chicken, pigs, fish, cattle, and buffaloes), grocery business, vehicle driving (harvest machine) and external work (labour work, civil service, and teaching).

**Table 1:** Income-generating activities amongst long-distance free-grazing duck farmers, paddy owners and duck transporters included in the study.

	Farmers (n = 44)	Paddy owners (n = 23)	Transporters (n = 17)
% of respondents who cultivate rice	43%	100%	35%
% of respondents who keep ducks	100%	17%	24%
% of respondents who name rice as primary source of income	7%	91%	6%
% of respondents who name ducks as primary source of income	89%	0%	12%
% of respondents who name transport as primary source of income	N/A	N/A	76%
% of respondents who name paddy rental as primary source of income	N/A	0	N/A
Median (maximum) number of different income-generating activities	3 (7)	4 (6)	3 (5)

N/A: not applicable

While all paddy owners reported letting their paddies to FGD farmers after most harvests, 65% (15/23) rated this income as “not important” and none rated it as “very important”. The sale of rice was the main source of income for most of the paddy owners (91%, 21/23). Around 39% (9/23) reported that they systematically give the money received from the farmers to “charities”, which refers to communal associations who take care of disadvantaged people, run ambulance services or maintain infrastructures such as roads and bridges. Twelve paddy owners provided detailed data on the income obtained from their rice paddies. The annual income per hectare from the sale of the rice and from the rice paddy rental ranged from 30 to 80 million VND (1,300 to 3,500 USD) and from 0.45 to 1.5 million VND (20 to 70 USD), respectively. On average, the latter represented 2.5% of the income generated by the former.

### Long-distance movement characteristics of free-grazing duck flocks

**Fig 1:**
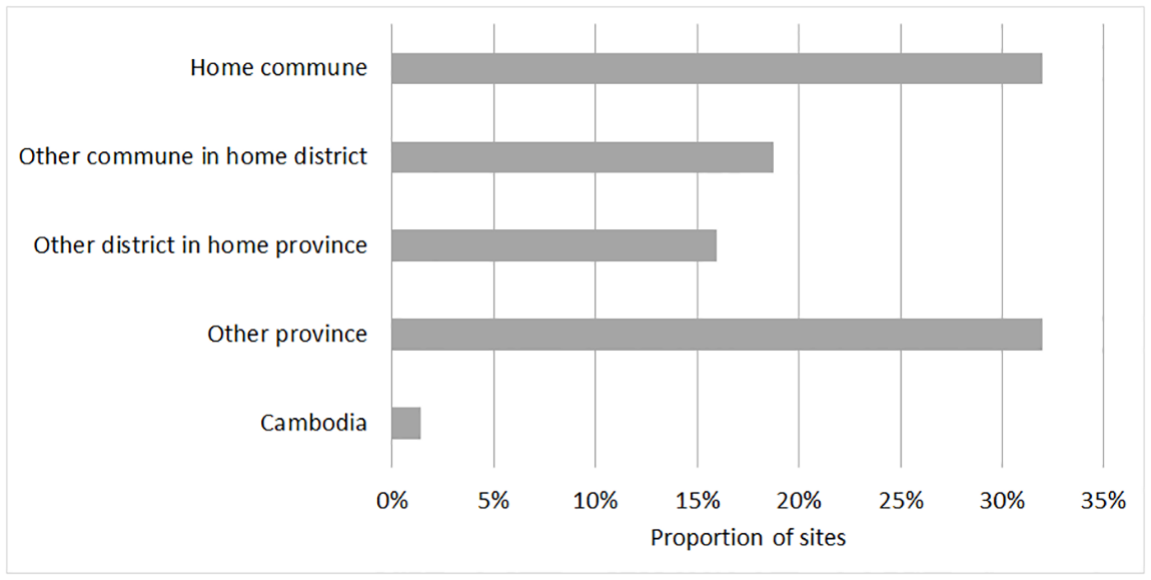
Location of scavenging sites (N = 219) used by FGD farmers relative to the location of their home village.

**Fig 2:**
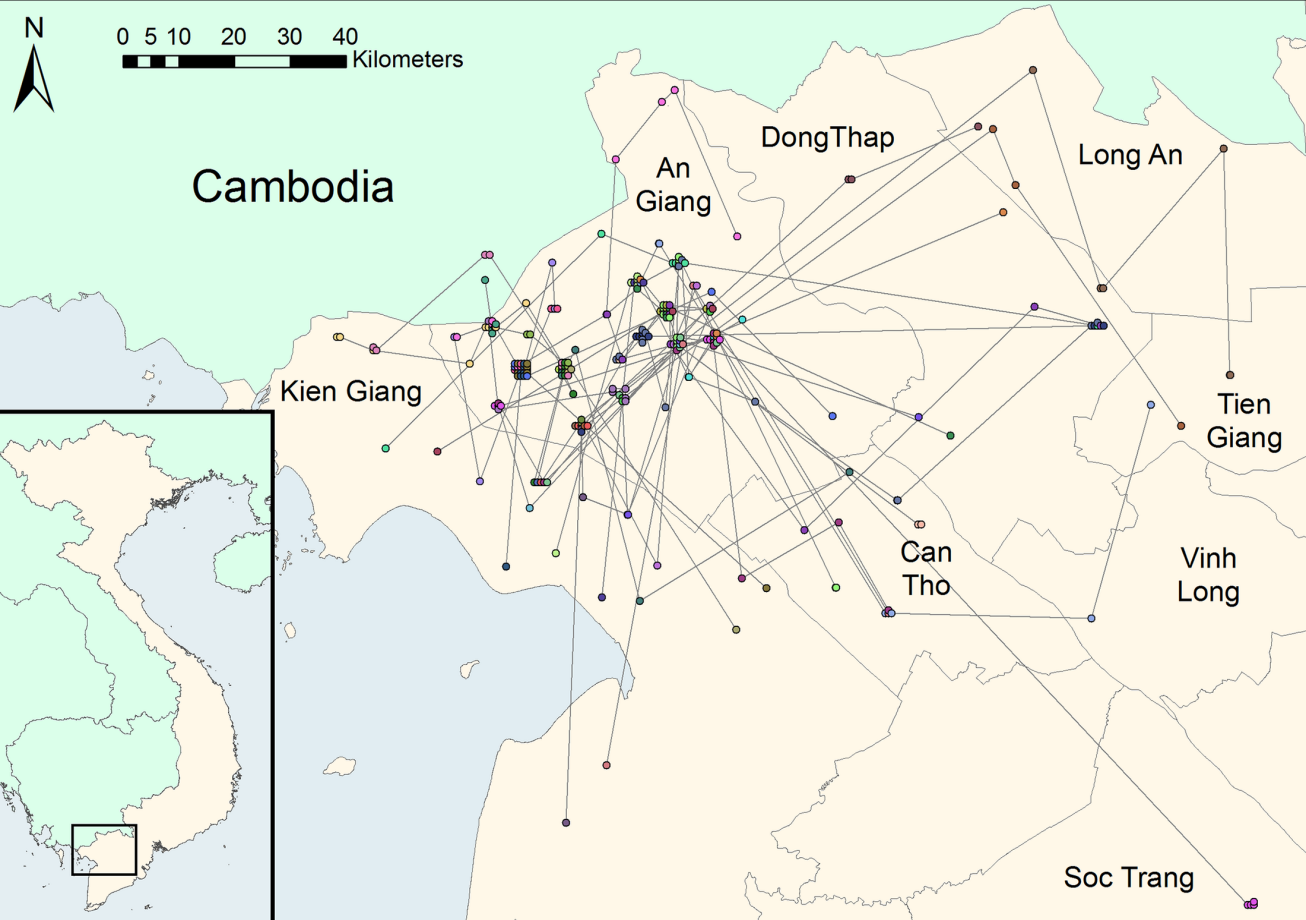
Geographical distribution of the 219 scavenging sites used by FGD farmers. The sites used by the same farmer are marked with the same colour and linked by lines representing the journeys between sites. This figure has been produced using ArcMap version 10.1 [31].

The location of the 219 unique sites reported by the 44 farmers during the interviews is shown on Figs 1 and 2. Among these sites, 146 (67%) were located in the home province of the reporting farmer, while most of the remaining sites were in other Vietnamese provinces of the MRD apart from 2 (1%) which were located in Cambodia, close to the Vietnamese border. The median distance between two successive journeys was 30 km (standard deviation: 16 km, range: 1–85). The total distance travelled by each flock between the date when it was purchased and the date of the survey ranged between 21 and 763 km (median 125 km with standard deviation 140 km). Three means of transport were used by the farmers to move their flocks between paddies: out of the 209 journeys for which information was available, 137 (66%) were by boat, 25 (12%) by truck and 47 (22%) by foot. All farmers but one transported their flock back to their home commune during its post-harvest season (i.e., two to three times a year) either to let their flock scavenge on their own paddies (43% (19/44) of farmers also owned rice paddies) or on hired paddies. Consequently, the rice harvest calendar in their home commune often informed the timing of journeys of these farmers. As the layer duck production cycle is relatively long (up to two years), FGD flocks return to their home commune up to six times during their life time. The ability to obtain access to rice paddies was the most common reason cited by farmers for choosing scavenging sites for their duck flocks (48%). Familiarity with the locality and its inhabitants accounted for 31% of the total and cost-benefit analyses for 10%. Other reasons mentioned by farmers were the distance between the site and their home, the quality of the paddies, and the ease of access.

### Contact patterns of FGD flocks

Four types of contacts between FGD flocks were identified by the stakeholders: (i) direct outdoors: two flocks accidentally mix during scavenging, on the waterways or in the night enclosures; (ii) indirect outdoors: two flocks use the same paddy sequentially during the same harvest season with a maximum of two days between the departure of the first flock and the arrival of the second; (iii) direct during transport: two or more flocks are transported together in the same vehicle to reduce the cost associated with the journey; and (iv) indirect during transport: two flocks are transported sequentially in the same vehicle without cleaning and disinfection of the vehicle between the two journeys. The nature and frequency of the contacts between FGD flocks, as reported by farmers and paddy owners, are summarised in Table 2. The frequency of reporting both direct and indirect contacts was not statistically significantly different between farmers and paddy owners. The occurrence of direct and indirect contacts between flocks was reported by 71% and 46% of stakeholders, respectively. The frequency of contacts was qualified as “intermediate” by the farmers, since direct and indirect contacts occurred on 40% and 13% of the paddies, respectively. Contacts between FGD flocks might also occur on the waterways. All farmers involved in the study used the canals surrounding the paddies for their ducks to wash off the mud accumulated during scavenging. While none of them reported any direct contact in waterways, all agreed that indirect contacts are likely to occur.

**Table 2:** Type and frequency of contacts between duck flocks reported by farmers, paddy owners, and duck transporters in the study.

Type of contact	% of paddy owners reporting that contacts happen (N = 19)	% of farmers reporting that contacts happen (N = 44)	% of sites where contacts happened at least once, as reported by farmers (N = 219)
Direct outdoors	74	70	40
Indirect outdoors	63	39 ^a^	13 ^a^
With wild waterfowl		55 ^b^	21 ^b^
With other wild birds		95 ^c^	73 ^c^
Direct during transport		9	

^a,b,c^: the difference between values with the same superscript was statistically significant (χ^2^ test, p-value < 0.05)

The perception in relation to shared use of vehicles by at least two flocks differed between farmers and transporters, in that the former reported sharing vehicles during 10% (16/162) of the journeys and the latter 31% (500/1600) (χ^2^ test, p-value < 0.01). Indirect contacts between FGD flocks were very frequent as transport vehicles are rarely cleaned and disinfected. Only three transporters (18%) reported washing the inside of their vehicle with water after each journey, while half of the interviewees reported cleaning their vehicle every six months or less often. Three transporters (18%) reported disinfecting their vehicle, using a commercial combination of glutaraldehyde and benzalkonium. The frequency of disinfection ranged from “after each journey” for one of them to “every other month” for the two others.

Additionally, it was mentioned that FGD flocks might be in contact with wild birds during scavenging. Because of their different role in avian influenza epidemiology, wild birds were categorised into wild waterfowl and other wild birds. As shown in Table 2, contacts with wild birds other than waterfowl were very frequent as they were reported by almost all farmers (95%) and for 73% of the sites. More than half of the farmers reported that their current flock had had contacts on at least one occasion with wild waterfowl (55%), but at a limited number of sites (21%) and mostly during the late rainy season.

### Risk factors for avian influenza A viruses

Out of the ten trucks from which fresh faecal samples were collected, two had at least one sample positive for the influenza A virus matrix gene. Due to field logistical constraints, only 29 flocks out of 44 were sampled. Nine of these (31%) had at least one pooled sample positive for the influenza A virus matrix gene. All samples (duck flocks and vehicles) were negative for H5 virus subtype.

Eight explanatory variables were associated with the influenza A status of the flock in the univariable analysis (Table 3). Following the variable selection process, three explanatory variables were retained in the final regression model: the location of the flock at the time of sampling (at home or not), the age of the ducks and the size of the flock were significantly associated with the circulation of influenza A viruses in the flock. The variance inflation factors did not indicate multicollinearity. The estimated odds ratios associated with each of these risk factors, as well as their 95% confidence interval (95%CI), are presented in Table 4. Notably, neither movement patterns (distances, type of transport, etc.) nor the frequency of contacts with wild birds or with other FGD flocks were retained as significant predictors of influenza A positivity of the flock.

**Table 3:** Variables statistically significantly associated with the detection of influenza A virus in univariable analysis (at p-value < 0.2 using a likelihood-ratio test).

**Categorical variables**				
**Variable**	**Categories**	**Number of flocks sampled**	**Number of flocks positive to influenza A**	**Likelihood ratio test p-value**
Location at time of sampling	At home	13	1	0.0097
	Not at home	16	8	
Number of flocks	Several flocks	15	2	0.030
	One flock only	14	7	
Consistency of the journey	Different journey each season	14	7	0.030
	Same journey each season	15	2	
**Continuous variables**				
**Variable**	**Mean (standard deviation) in flocks negative to influenza A**		**Mean (standard deviation) in flocks positive to influenza A**	**Likelihood ratio test p-value**
Size of the flock	4,023 (3,986)		2,389 (639)	0.13
Number of other income-generating activities	2 (2)		1 (2)	0.16
Age of the flock (months)	9 (3)		6 (2)	0.0064
Duration since last journey (days)	30 (30)		12 (11)	0.041

**Table 4:** Results of the multivariable logistic regression with the detection of influenza A virus as the outcome variable.

Variable	Categories	Odds ratio	
		Point estimate	95%CI
Location at time of sampling	At home	*Ref*	
	Not at home	17.0	1.77–440
Age of the flock (months)		0.57	0.23–0.90
Size of the flock (x 500 ducks)		0.81	0.49–0.99

## Discussion

### Description of farming system

Although the use of the free-grazing duck production system has been on the decline in South-East Asia for the last three decades as a result of intensification of agricultural production, increasing cost of labour and increasing pesticide use [34], the FGD system is still widely practised in at least four countries: China, Indonesia, Thailand and Vietnam [9, 18, 35]. The objectives of this study were to describe the long-distance FGD farming system practiced in South Vietnam, to assess the level of circulation of influenza A viruses in free-grazing duck flocks, and to identify potential risk factors for influenza A infection in our study population.

Generally, the description of the system revealed a number of flaws related to biosecurity practices in the free grazing duck production, which represent opportunities for the circulation of pathogens between FGD flocks. The long duration of the production cycle (up to two years according to our results) and the complexity of the production system are also likely to be important factors in the epidemiology of avian diseases in general, and HPAI in particular. The analysis of the flocks’ movements showed that these flocks are very mobile within the MRD, as indicated in previous studies [23, 36]. Trade of live poultry from Vietnam to neighbouring Cambodia has been described by Van Kerkhove, Vong [37]. In our study, only a couple of farmers reported occasionally using scavenging sites located in Cambodia. They crossed the border with their flocks by foot across the paddies and did not venture further than 5 km into Cambodian territory. However in a study by Nguyen, Siembieda [38], some FGD flocks were regularly herded further into Cambodia for scavenging, before being re-introduced to Vietnam, as Cambodia has a lower duck density and rice paddies are often available in Cambodia at times when none are available in Vietnam due to different rice calendars. Wei, Lin [39] showed that virus strains isolated in Cambodia and Vietnam presented a high level of homology, suggesting that poultry movements across the border and/or wild bird migration create epidemiological links between the two countries.

By boat was the most common transport means used by duck farmers due to the well-developed canal network, in contrast to the poor road network in our study area. Contacts between duck flocks during transport were reported to happen regularly. In addition, cleaning and disinfection of the vehicles was found to be insufficient, increasing the risk of pathogen transmission between flocks, as previously suggested [8, 40, 41]. Lack of hygiene in transport represents a substantial breach of biosecurity practices, and so does the range of other contacts were reported by the duck farmers, mainly in the field during grazing, with other domestic ducks and with wildlife. A similar survey conducted in 2007 in four provinces of the MRD reported lower contact rates between FGD flocks [23]. This might be explained by the fact that their sample included not only layer duck farmers but also meat duck farmers, whose farming practices may differ. Most of the direct contacts between FGD flocks as reported in our study were accidental. Indeed, within the paddy rental system, farmers pay a fee to the paddy owner which allows exclusive use to the paddy field for the duration agreed upon. Even if a small number of farmers use nets to prevent their flock from wandering beyond the paddy’s perimeter, most of the interviewed farmers do not use them and instead prevent the mixing with flocks scavenging on neighbouring paddies via manual herding only. As a consequence, frequent mixing occurs where a small number of ducks from a flock escape and temporarily join other flocks. The paddy rental system sometimes co-exists with another paddy management system, in which rice paddies cultivated by farmers from a given village are available after harvest for any duck farmer from the village. Therefore, these paddies are sometimes used by both short- and long-distance FGD flocks. According to the first author’s personal observations, as the density of ducks tends to be higher than on privately rented paddies, contacts are more frequent in the community paddy system. In agreement with the study from Henning, Henning (23), none of the farmers surveyed in our study reported contacts between duck flocks and chickens. This seems to be associated with the geographically separate scavenging patterns of these animals, with chickens wandering around the villages and long-distance FGD flocks scavenging on the paddies and being kept at night in enclosures next to the paddies, i.e., away from the villages. Conversely, contacts between chicken and short-distance FGDs or backyard ducks and their epidemiological consequences have been reported in the region [42, 43].

Overall, the interviews with the stakeholders showed that the long-distance FGD production system is characterised by a highly connected contact network, where the connections of a farmer to rice paddy owners, transporters, traders and other farmers are critical for the success of their business. For instance, familiarity with the paddy owners accounted for a third of the reasons influencing farmers’ choice of scavenging sites. Annual variation in rainfall and floods, intensification of rice cultivation, increasing urbanisation as well as competition between duck farmers limit the availability of post-harvest rice paddies for duck scavenging. In this respect, farmers with established experience and those who were well connected appeared more successful at obtaining sufficient access to rice paddies all year round and thereby being able to reduce the costs of long-distance journeys across the river delta. From the paddy owners’ point of view, two thirds of the interviewees reported that the income from the paddy rental was not important for their business. The benefits of renting out the paddies are mainly in kind from ducks feeding on a range of rice pests such as golden snails [44] and fertilising the paddies. Some paddy owners also reported renting their paddies “out of generosity” to the FGD farmers. These results suggest that HPAI management measures in Vietnam should take into account not only the farmers but also the other stakeholders involved in the system, such as paddy owners and transporters, as previously highlighted for Indonesia [20]. Further, the description of the socio-economic characteristics of these stakeholders provide useful elements to understand their behaviours in the context of HPAI management [45].

### Circulation of influenza A viruses

In our study, 31% of the flocks tested positive for influenza A viruses, indicating a high level of circulation of these viruses. Significant predictors of the influenza A status of the flocks were the age and size of the flocks as well as the location at the time of sampling relative to the home location. No flocks tested positive for H5 subtype viruses. Analyses of outbreak data have highlighted the seasonality of H5N1 outbreaks in the South of Vietnam, with a marked high-risk season between December and February [4, 46]. However, a recent study in the MRD showed that 14 and 18% of duck flocks tested positive for H5 antigen during low- and high-risk periods, respectively [47], suggesting an all-year round circulation of HPAI viruses in poultry even in the absence of reported outbreaks. The absence of H5 positive flocks in our study may be due to absence of virus circulation at the time of the survey, sampling bias or small sample size, as discussed under study limitations.

Poultry vaccination programmes against HPAI viruses have been implemented in Vietnam since 2005. In our study, all free-grazing duck flocks were vaccinated, as previously reported in other provinces of the MRD [18, 36]. It has previously been shown that in Vietnam duck flocks that received no HPAI vaccination or only one injection were at a higher risk of HPAI outbreak compared to flocks with two or more injections [43]. In our study, the number of vaccine injections was not associated with the influenza A positivity which might be explained by the absence of cross-protection offered by the HPAI vaccine against other influenza A virus serotypes [48], or by the low statistical power of our study as discussed under study limitations below. On the other hand, some studies have suggested that vaccination may facilitate the silent spread of the H5N1 virus due to partial vaccination coverage at herd-level, partial matching of wild and vaccine strains and difficulty to detect outbreaks among vaccinated animals [6, 49, 50]. This supports our hypothesis that FGD may play an important role in maintaining HPAI virus circulation. A veterinary certificate, documenting appropriate HPAI vaccination, is theoretically required to move poultry between communes in the country, both for trade and scavenging purposes. These certificates are provided and checked by Animal Quarantine Stations, located on the main roads connecting districts and provinces. But a large number of movements are very likely not captured by the official records, as movements of poultry by boat or by foot across paddies are common (88% of journeys in our study).

According to the multivariable analysis, flocks which were not in their home commune at the time of sampling were more likely to be positive for influenza A infection than flocks which were at home. Also, influenza A positive flocks had travelled more recently than negative flocks as shown by the univariable analysis. Visiting flocks and flocks which had travelled recently have experienced stressful transport conditions, which may have affected the immunity of the birds and therefore explain the increased odds of influenza A infection, as reported elsewhere [22]. Farmers owning larger flocks, or more than one duck flock, were less likely to have influenza A infection detected in their flocks. Biosecurity measures (such as visitors entering the pens and sharing transport vehicles for instance) are generally better implemented in larger-scale duck farms, and may explain the lower risk of influenza A infection compared to smaller farms. Breaches of biosecurity, such as people visiting the flocks, were identified as risk factors in other studies [22, 43, 51]. The age of the flock was retained as a protective factor in our multivariable analysis, with older flocks having a lower odds of influenza A infection than younger ones. This is a similar finding to the report by Henning, Henning (43), where ducks less than two months old had higher odds of infection by HPAI than older ducks. A better protective immunity of older flocks through repeated exposure to influenza A virus, naturally or via vaccination, may explain this association.

Avian species present on the rice fields at the time of the survey included egret, heron and pond heron species which have been reported to be susceptible to HPAI infection [52] but there appears to not have been any direct exposure to domestic poultry apart from FGDs (A. Meyer, personal observations). Also, our study did not show that the contacts reported by the farmers with other domestic ducks or wild birds were associated with risk of influenza A infection. Again, it needs to be noted that the study had a low statistical power and there may have been sampling bias. This result is consistent with the findings from a study in Indonesia, where the presence of other animals (ducks, chickens and wild birds) in the enclosures was not associated with H5 seroconversion in ducks [53]. However, it does not corroborate other studies that showed FGD flocks sharing scavenging locations with other poultry, including ducks from other farms, were at increased risk of influenza outbreaks [22, 43].

### Study limitations

The main difficulty when working on long-distance FGD flocks is their recruitment. Some previous studies based their sampling on local poultry flock registers from local veterinary services (see for example Beaudoin, Kitikoon (22)). However, such an approach does not allow for the recruitment of visiting duck flocks, which would mean preventing the sample to fully represent the long-distance FGD population present within the study area at the time of a survey. As described in other recent publications [23], the convenience sampling strategy used in the present study enabled us to overcome this difficulty.

It is difficult to assess whether the long-distance FGD population that was present at the time of the study was representative of the whole region as no large-scale study has tried to describe the spatio-temporal distribution of the long-distance FGD flocks so far. The province of An Giang has a duck density of 12 heads per hectare of rice fields which is comparable to the average density in the MRD (Department of Animal Health, personal communication, 2016) and the production system described in our study presents many common features with those reported for other provinces of the MRD, as discussed above. Therefore, the province of An Giang can be considered as a rice and duck producing region representative of the MRD. However, it has to be kept in mind that the province has a proportionally higher surface of rice fields than the rest of the MRD (73% of the province area, compared to an average 45% in the other provinces)[54], which could result in a higher density of visiting FGD flocks and therefore a higher amount of direct and indirect contacts between FGD flocks in that province.

The use of questionnaires probably introduced some bias to the data collected from the different stakeholders: recall bias as well as bias due to social acceptability of the answers might have occurred. Even though the questions related to past journeys and activities focused on those performed with the current flock, some parts of the journey will have been already several months old, potentially leading to recall biases regarding different parameters such as duration of the transport and occurrence of contacts with other flocks for instance. Last, technical constraints limited the number of stakeholders recruited in the biological study (only 29 out of 44 duck flocks were sampled as initially foreseen). Indeed, long-distance FGD flocks are often transported to different districts and provinces (see Fig 1) to follow rice harvest cycles. This explains why 15 flocks moved after the interview and were therefore not available to be sampled by the veterinary services. As a consequence, the multivariable regression analysis component of the study had low statistical power and only three variables were identified as risk factors in the final model.

## Conclusions

The free-grazing duck production system is characterised by intense and diverse contacts between duck flocks as well as long-distance journeys potentially leading to the spread of pathogens. However, it provides a major source of income for a large number of stakeholders in many countries in South-East Asia. It is therefore paramount to examine this system carefully, assess its contribution to the maintenance of influenza viruses and generate realistic and acceptable recommendations for risk management without threatening the livelihood of thousands of farmers. This work provides a detailed description of the stakeholders involved in this system and of factors that contribute to sustaining local and regional circulation of influenza viruses. The findings can be used to develop risk assessment models of influenza virus spread to develop better biosecurity practices ultimately leading to better animal health, sustainable animal production and reliable income for the farmers involved.

## Supporting information

S1 File Questionnaire for individual interviews with long-distance free grazing duck farmers. (PDF)

S2 File Questionnaire for individual interviews with rice paddy owners. (PDF)

S3 File Questionnaire for individual interviews with transporters of free grazing ducks. (PDF)

S4 File Dataset of long-distance free grazing duck farmers. (TXT)

S5 File Dataset of sites used for free grazing. (TXT)

S6 File Dataset of rice paddy owners. (TXT)

S7 File Dataset of transporters of free grazing ducks. (TXT)

## References

[1] Brown IH. Summary of avian influenza activity in Europe, Asia, and Africa, 2006–2009. Avian diseases. 2010;54(s1):187–93. 20521631 10.1637/8949-053109-Reg.1

[2] Alexander DJ. Summary of avian influenza activity in Europe, Asia, Africa, and Australasia, 2002–2006. Avian diseases. 2007;51(s1):161–6. 17494548 10.1637/7602-041306R.1

[3] World Animal Health Information System [Internet]. World Organisation for Animal Health. 2016 [cited 12/12/2016]. www.oie.int/wahis_2.

[4] Pfeiffer DU, Minh PQ, Martin V, Epprecht M, Otte MJ. An analysis of the spatial and temporal patterns of highly pathogenic avian influenza occurrence in Vietnam using national surveillance data. The Veterinary Journal. 2007;174(2):302–9. 10.1016/j.tvjl.2007.05.010. 17604193

[5] Minh PQ. Epidemiological studies of Highly Pathogenic Avian Influenza in Vietnam. Palmerston, New-Zealand: Massey University; 2010.

[6] Nguyen V. The epidemiology of avian influenza in the Mekong River Delta of Vietnam. Palmerston, New-Zealand: Massey University; 2013.

[7] Gilbert M, Newman SH, Takekawa JY, Loth L, Biradar C, Prosser DJ, et al Flying over an infected landscape: distribution of highly pathogenic avian influenza H5N1 risk in South Asia and satellite tracking of wild waterfowl. EcoHealth. 2010;7(4):448–58. 10.1007/s10393-010-0672-8 21267626 PMC3166606

[8] Paul M, Tavornpanich S, Abrial D, Gasqui P, Charras-Garrido M, Thanapongtharm W, et al Anthropogenic factors and the risk of highly pathogenic avian influenza H5N1: prospects from a spatial-based model. Veterinary research. 2010;41(3):28 10.1051/vetres/2009076 20003910 PMC2821766

[9] Gilbert M, Chaitaweesub P, Parakamawongsa T, Premashthira S, Tiensin T, Kalpravidh W, et al Free-grazing ducks and highly pathogenic avian influenza, Thailand. Emerg Infect Dis. 2006;12(2):227–34. Epub 2006/02/24. 10.3201/eid1202.050640 . 16494747 PMC3373083

[10] Gilbert M, Pfeiffer DU. Risk factor modelling of the spatio-temporal patterns of highly pathogenic avian influenza (HPAIV) H5N1: a review. Spatial and spatio-temporal epidemiology. 2012;3(3):173–83. Epub 2012/07/04. 10.1016/j.sste.2012.01.002 . 22749203 PMC3389348

[11] Vergne T, Paul MC, Chaengprachak W, Durand B, Gilbert M, Dufour B, et al Zero-inflated models for identifying disease risk factors when case detection is imperfect: application to highly pathogenic avian influenza H5N1 in Thailand. Prev Vet Med. 2014;114(1):28–36. Epub 2014/01/30. 10.1016/j.prevetmed.2014.01.011 . 24472215

[12] Hulse-Post DJ, Sturm-Ramirez KM, Humberd J, Seiler P, Govorkova EA, Krauss S, et al Role of domestic ducks in the propagation and biological evolution of highly pathogenic H5N1 influenza viruses in Asia. Proc Natl Acad Sci U S A. 2005;102(30):10682–7. Epub 2005/07/21. 10.1073/pnas.0504662102 . 16030144 PMC1180796

[13] Desvaux S, Grosbois V, Pham TT, Fenwick S, Tollis S, Pham NH, et al Risk factors of highly pathogenic avian influenza H5N1 occurrence at the village and farm levels in the Red River Delta Region in Vietnam. Transbound Emerg Dis. 2011;58(6):492–502. Epub 2011/05/07. 10.1111/j.1865-1682.2011.01227.x . 21545692

[14] Sturm-Ramirez KM, Hulse-Post DJ, Govorkova EA, Humberd J, Seiler P, Puthavathana P, et al Are ducks contributing to the endemicity of highly pathogenic H5N1 influenza virus in Asia? J Virol. 2005;79(17):11269–79. Epub 2005/08/17. 10.1128/JVI.79.17.11269-11279.2005 . 16103179 PMC1193583

[15] Webster RG. Wet markets—a continuing source of severe acute respiratory syndrome and influenza? The Lancet. 2004;363(9404):234–6. 10.1016/S0140-6736(03)15329-9. PMC7112390 14738798

[16] Fournié G, Guitian J, Desvaux S, Mangtani P, Ly S, Cong VC, et al Identifying live bird markets with the potential to act as reservoirs of avian influenza A (H5N1) virus: a survey in northern Viet Nam and Cambodia. PLoS One. 2012;7(6):e37986 10.1371/journal.pone.0037986 22675502 PMC3366999

[17] Desvaux S, Vu DT, Phan DT, Pham TTH. A general review and a description of the poultry production in Vietnam. PRISE, a Research Consortium on Risks associated with Livestock Intensification, 2008 2008. Report No.

[18] Henning J, Henning K, Vu L, Yulianto D, Meers J. The role of moving duck flocks in the spread of Highly Pathogenic Avian Influenza virus in Vietnam and Indonesia. Proceedings of the 12th Symposium of the International Society for Veterinary Epidemiology and Economics; Durban, South Africa2009. p. 30.

[19] Le HP. Poultry Waste Management in the An Giang province Of the Mekong Delta. Food and Agriculture Organization of the United Nations, 2010 2010. Report No.

[20] Henning J, Pfeiffer DU, Stevenson M, Yulianto D, Priyono W, Meers J. Who Is Spreading Avian Influenza in the Moving Duck Flock Farming Network of Indonesia? PLoS One. 2016;11(3):e0152123 10.1371/journal.pone.0152123 27019344 PMC4809517

[21] Bui XM. Duck farming systems and avian influenza in the Mekong Delta of Viet Nam. Rome, Italy: Food and Agriculture Organization of the United Nations, 2010.

[22] Beaudoin AL, Kitikoon P, Schreiner PJ, Singer RS, Sasipreeyajan J, Amonsin A, et al Risk factors for exposure to influenza a viruses, including subtype H5 viruses, in Thai free-grazing ducks. Transbound Emerg Dis. 2014;61(4):362–74. Epub 2013/01/03. 10.1111/tbed.12043 . 23279757

[23] Henning J, Henning K, Ngo T, Nguyen T, Le T, Meers J. Characteristics of two duck farming systems in the Mekong Delta of Viet Nam: stationary flocks and moving flocks, and their potential relevance to the spread of highly pathogenic avian influenza. Trop Anim Health Pro. 2013;45(3):837–48. Epub 2012/10/23. 10.1007/s11250-012-0296-9 . 23086602

[24] Sturm-Ramirez KM, Ellis T, Bousfield B, Bissett L, Dyrting K, Rehg JE, et al Reemerging H5N1 influenza viruses in Hong Kong in 2002 are highly pathogenic to ducks. Journal of virology. 2004;78(9):4892–901. 10.1128/JVI.78.9.4892-4901.2004 15078970 PMC387679

[25] Ellis TM, Barry Bousfield R, Bissett LA, Dyrting KC, Luk GS, Tsim S, et al Investigation of outbreaks of highly pathogenic H5N1 avian influenza in waterfowl and wild birds in Hong Kong in late 2002. Avian Pathology. 2004;33(5):492–505. 10.1080/03079450400003601 15545029

[26] Spackman E, Senne DA, Myers T, Bulaga LL, Garber LP, Perdue ML, et al Development of a real-time reverse transcriptase PCR assay for type A influenza virus and the avian H5 and H7 hemagglutinin subtypes. Journal of clinical microbiology. 2002;40(9):3256–60. 10.1128/JCM.40.9.3256-3260.2002 12202562 PMC130722

[27] Burnham KP, Anderson DR. Model Selection and Inference: A Practical Information-theoretic Approach: Springer; 1998 353 p.

[28] Montgomery DC, Peck EA, Vining GG. Introduction to linear regression analysis: John Wiley & Sons; 2015.

[29] R Core Team. R: A Language and Environment for Statistical Computing. Vienna, Austria: R Foundation for Statistical Computing; 2014.

[30] Ripley B, Venables B, Bates DM, Hornik K, Gebhardt A, Firth D, et al. Package ‘MASS’. CRAN Repository http://cran.r-project.org/web/packages/MASS/MASS.pdf. 2013.

[31] ESRI. ArcGIS Desktop: Release 10. Redlands, CA: Environmental Systems Research Institute 2011.

[32] NAVETCO. Vaccines manufactured at NAVETCO for poultry: Navet-Vifluvac 2014 [September 30, 2016]. http://www.navetco.com.vn/en/product/navet-vifluvac.

[33] Merial. H5N1 reassortant avian influenza virus vaccine, inactivated. 2012 [September 30, 2016]. http://advance-animalbd.com/imgs/Poultry/Biologicals/RE6.pdf.

[34] Tai C, Tai J-JL. Future Prospects of Duck Production in Asia. The Journal of Poultry Science. 2001;38(1):99–112. 10.2141/jpsa.38.99

[35] Gilbert M, Golding N, Zhou H, Wint GRW, Robinson TP, Tatem AJ, et al Predicting the risk of avian influenza A H7N9 infection in live-poultry markets across Asia. Nat Commun. 2014;5 10.1038/ncomms5116 24937647 PMC4061699

[36] Minh PQ, Stevenson M, Schauer B, Morris R, Quy TD. A description of the management of itinerant grazing ducks in the Mekong River Delta of Vietnam. Prev Vet Med. 2010;94(1):101–7. 20015558 10.1016/j.prevetmed.2009.11.011

[37] Van Kerkhove MD, Vong S, Guitian J, Holl D, Mangtani P, San S, et al Poultry movement networks in Cambodia: implications for surveillance and control of highly pathogenic avian influenza (HPAI/H5N1). Vaccine. 2009;27(45):6345–52. 10.1016/j.vaccine.2009.05.004 19840671

[38] Nguyen TTT, Siembieda J, Le HP, Pham TL, Nguyen PO. Internal report on the movement of ducks cross-border for trading and production purposes. Food and Agriculture Organization of the United Nations—Vietnamese representation, 2012.

[39] Wei K, Lin Y, Xie D. Evolutionary and Ecological Dynamics of Transboundary Disease Caused by H5N1 Virus in Southeast Asia. Transbound Emerg Dis. 2015;62(3):315–27. Epub 2013/08/21. 10.1111/tbed.12147 . 23952973

[40] Yupiana Y, de Vlas SJ, Adnan NM, Richardus JH. Risk factors of poultry outbreaks and human cases of H5N1 avian influenza virus infection in West Java Province, Indonesia. International Journal of Infectious Diseases. 2010;14(9):e800–e5. 10.1016/j.ijid.2010.03.014 20637674

[41] Kilpatrick AM, Chmura AA, Gibbons DW, Fleischer RC, Marra PP, Daszak P. Predicting the global spread of H5N1 avian influenza. Proceedings of the National Academy of Sciences. 2006;103(51):19368–73. 10.1073/pnas.0609227103 PMC1748232 17158217

[42] Henning J, Henning K, Morton J, Long T, Nguyen T, Le T, et al Highly pathogenic avian influenza (H5N1) in ducks and in-contact chickens in backyard and smallholder commercial duck farms in Viet Nam. Prev Vet Med. 2011;101(3–4):229–40. 10.1016/j.prevetmed.2010.05.016. 20594603

[43] Henning KA, Henning J, Morton J, Long NT, Ha NT, Meers J. Farm- and flock-level risk factors associated with Highly Pathogenic Avian Influenza outbreaks on small holder duck and chicken farms in the Mekong Delta of Viet Nam. Prev Vet Med. 2009;91(2–4):179–88. Epub 2009/07/08. 10.1016/j.prevetmed.2009.05.027 . 19581011

[44] Adzitey F, Adzitey S. Duck production: has a potential to reduce poverty among rural households in Asian communities–a review. J World’s Poult Res. 2011;1:7–10.

[45] Rich KM, Perry BD. The economic and poverty impacts of animal diseases in developing countries: New roles, new demands for economics and epidemiology. Preventive Veterinary Medicine. 2011;101(3–4):133–47. 10.1016/j.prevetmed.2010.08.002. 20828844

[46] Minh PQ, Morris RS, Schauer B, Stevenson M, Benschop J, Nam HV, et al Spatio-temporal epidemiology of highly pathogenic avian influenza outbreaks in the two deltas of Vietnam during 2003–2007. Prev Vet Med. 2009;89(1–2):16–24. Epub 2009/02/24. 10.1016/j.prevetmed.2009.01.004 . 19232765

[47] Nguyen LV, Stevenson M, Schauer B, Nguyen D, Tran Q, Tien T, et al Descriptive results of a prospective cohort study of avian influenza in the Mekong River Delta of Viet Nam. Transboundary and emerging diseases. 2014;61(6):511–25. 10.1111/tbed.12055 23331425

[48] Suarez DL, Schultz-Cherry S. Immunology of avian influenza virus: a review. Developmental & Comparative Immunology. 2000;24(2–3):269–83. 10.1016/S0145-305X(99)00078-6. 10717293

[49] Savill NJ, St Rose SG, Keeling MJ, Woolhouse MEJ. Silent spread of H5N1 in vaccinated poultry. Nature. 2006;442(7104):757-. http://www.nature.com/nature/journal/v442/n7104/suppinfo/442757a_S1.html. 10.1038/442757a 16915278

[50] Vergne T, Grosbois V, Jobre Y, Saad A, El Nabi AA, Galal S, et al Avian influenza vaccination of poultry and passive case reporting, Egypt. Emerg Infect Dis. 2012;18(12):2076–8. Epub 2012/11/23. 10.3201/eid1812.120616 . 23171740 PMC3557869

[51] Fasina FO, Rivas AL, Bisschop SP, Stegeman AJ, Hernandez JA. Identification of risk factors associated with highly pathogenic avian influenza H5N1 virus infection in poultry farms, in Nigeria during the epidemic of 2006–2007. Preventive veterinary medicine. 2011;98(2):204–8. 21146235 10.1016/j.prevetmed.2010.11.007

[52] Siengsanan J, Chaichoune K, Phonaknguen R, Sariya L, Prompiram P, Kocharin W, et al Comparison of outbreaks of H5N1 highly pathogenic avian influenza in wild birds and poultry in Thailand. Journal of wildlife diseases. 2009;45(3):740–7. 10.7589/0090-3558-45.3.740 19617484

[53] Henning J, Morton JM, Wibawa H, Yulianto D, Usman TB, Prijono W, et al Incidence and risk factors for H5 highly pathogenic avian influenza infection in flocks of apparently clinically healthy ducks. Epidemiol Infect. 2013;141(2):390–401. Epub 2012/06/13. 10.1017/S0950268812001100 . 22687557

[54] GSO. 2011 Rural, Agricultural and Fishery Census. General Statistics Office of Vietnam, 2011.

