# Supplementary material for: Movement and contact patterns of long-distance free-grazing ducks and avian influenza persistence in Vietnam
Source: PLoS One. 2017 Jun 20;12(6):e0178241. doi: 10.1371/journal.pone.0178241 (PMC5478089; doi:10.1371/journal.pone.0178241)
Supplement: S1 File — (PDF) [file pone.0178241.s001.pdf]

Questionnaire for individual interviews with long-distance free grazing duck farmers

Location where the interview is conducted:
District: …………………..
Commune: ………………

Sentences in italics are instructions for the interviewer.

1. Identification of the farm

1.1. What are your name and surname?
……………………………………………………………………………….

1.2. Where do you live? *This refers to the permanent home of the farmer*.
Province: …………………
District: …………………..
Commune: ………………
Village: ……………………

1.3. How old are you? *Record the age of the farmer in years*.
………

1.4. Which ethnic group do you belong to?

☐ Kinh
☐ Khmer
☐ Mường
☐ Hoa
☐ Chăm
☐ Other…...

1.5. How many people belong to your household?
………….

2. Characteristics of the farm

2.1. *Ask the farmer to rank by income the productions and activities in which his/her household is involved. Ask him/her to think about the income over the last 12 months and record the rank number in the corresponding boxes*.

☐Rice
☐Other crops
☐Chicken
☐Ducks (common ducks or Muscovy ducks)
☐Geese
☐Pigs
☐Fish
☐Other: ……………………….

2.2. For how many years have you been keeping ducks?
………. years

2.3. Do you own more than one duck flock at the same time?

☐No
☐ Yes

3. Characteristics of the long-distance free grazing duck flock

For the remainder of the questions, tell the farmer that the questions refer to the long-distance free grazing flock he/she currently owns. If he/she owns several flock, ask him/her to refer to the oldest flock.

3.1. What is the size of your duck flock? *Record the current number of ducks in the flock*.
…………..
3.2. What is the age of your duck flock? *Record the current age of the ducks in days*.
……………
3.3. What type of ducks do you own?
☐Meat ducks
☐Layer ducks
☐Both
3.4. Which duck breed is your flock?
………………
3.5. How old were the ducks from your flock when you purchased them? *Record the age at purchase in days*.
……………….
3.6. Where did you purchase them?
☐Other farmer
☐Itinerant poultry trader
☐Market
☐Hatchery
☐Other: ……………………….

4. Flock management and travelling
4.1. Where do you intend to sell this flock?
☐ Market
☐ Poultry trader
☐ Slaughterhouse
☐ Other: ……………………………………………………………………..
4.2. On average, what is the age of the duck flock when you sell them? *Record the age at sale in months*.
………………………
4.3. Do you conduct the same journey each time?
☐No
☐Yes, same sites, but in a different order
☐Yes, same sites in the same order
4.4. *[Only if 4.3 is "No" or "Yes, same sites, but in a different order"]* Which factors do you take into account when deciding which free grazing sites you will visit?
……
……
4.5. How many times per year do you go back to your own commune for the free grazing?
………………………………

In sections 5 to 7, the term "site" refers to an individual rice paddy used for duck free grazing by the farmer.

5. Movement pattern of the current duck flock
In this section, ask the farmer to identify each of the sites already visited with his/her flock on the maps provided, included the current site. Mark the locations on the map with numbers in chronological order and record the answers to the following questions in the table at the end of the document using the same identification numbers. You should also identify the sites where the flock was kept in confinement.
For each site already visited (including the current site):
5.1. In which province, district and commune is this site located?

5.2. How long did your flock stay on that site? *Record the duration in days*.
5.3. Had the site already been used by another flock during the same harvest season before you arrive?

6. Contacts between the duck flock and other animals

Record the answers of this section in the same table.

For each site already visited (including the current site):

6.1. How many times did your flock have direct contacts with other domestic ducks when scavenging on that site (or in the waterways or in the night pens)?
6.2. Did your flock have contacts with poultry other than ducks when staying on that site (or in the waterways or in the night pens)?
6.3. Did your flock have contacts with wild waterfowl when staying on that site (or in the waterways or in the night pens)?
6.4. Did your flock have contacts with other wild birds when staying on that site (or in the waterways or in the night pens)?

7. Transport of the ducks between the different locations.

Record the answers of this section in the same table. Please make sure to record the details corresponding to the journey made to LEAVE the site on the line of each site, not the journey to ARRIVE on the site.

For each journey conducted to transport the flock to the sites listed in the table:

7.1. What type of transport did you use to move your flock?
7.2. How many duck flocks belonging to other farmers were present in the same vehicle? *Record N/A for journeys made by foot*.

8. Flock health management

8.1. How do you usually dispose of carcasses?

☐Burning
☐Burial
☐Disposal in environment
☐Consumption
☐Other…………….

8.2. Is your flock vaccinated against HPAI?

☐No
☐Yes, one injection
☐Yes, two injections
☐Yes, three injections or more

8.3. *[Only if 8.2 is not "No"]* How long ago was the last HPAI vaccine administered to your flock?
…………….

8.4. Is your flock vaccinated against other diseases?

☐Yes, specify diseases: …………………………..
☐No

Site ID	Grazing site					Contacts			Journey to LEAVE that site				
	Province	District	Commune	Duration (days)	Previously use (Y/N)	Free-grazing ducks (No. of contacts)	Other poultry (Y/N)	Wild birds (Y/N)	Type (foot, truck, boat, bike)	Duration (hours)	Length (km)	Other flocks (No. present)	Price (VND)
