# Supplementary material for: Movement and contact patterns of long-distance free-grazing ducks and avian influenza persistence in Vietnam
Source: PLoS One. 2017 Jun 20;12(6):e0178241. doi: 10.1371/journal.pone.0178241 (PMC5478089; doi:10.1371/journal.pone.0178241)
Supplement: S2 File — (PDF) [file pone.0178241.s002.pdf]

Questionnaire for individual interviews with rice paddy owners

Location where the interview is conducted:
District: ……………………
Commune: ………………

Sentences in italics are instructions for the interviewer.

1. Identification of the farm

1.1. What are your name and surname?
……………………………………………………………………………

1.2. Where do you live? *This refers to the permanent home of the respondent.*
Province: …………………
District: ……………………
Commune: ………………
Village: ……………………

1.3. How old are you? *Record the age of the respondent in years*.
………

1.4. Which ethnic group do you belong to?
☐Kinh
☐Khmer
☐Mường
☐Hoa
☐Chăm
☐Other: ……………………………………………

1.5. How many people belong to in your household?
…………

2. Characteristics of the farm

2.1. *Ask the respondent to rank by income the productions and activities in which his/her household is involved. Ask him/her to think about the production of the last 12 months and record the rank number in the corresponding boxes*.
☐Rice
☐Other crops
☐Chicken
☐Ducks (common ducks or Muscovy ducks)
☐Geese
☐Pigs
☐Fish
☐Rental of rice paddies to duck farmers
☐Other: ………………………..

2.2. How important is the income generated by renting the paddies to other farmers for their ducks?
☐Very important
☐Important
☐Not important

3. Characteristics of the field rental

3.1. What is the surface of paddies that you let to free grazing duck farmers? *Record the surfaces in công*.

Paddies with two rice cycles ………….

Paddies with three rice cycles ………….

3.2. How much income do you make per year by selling the rice produced on your paddies? *Record the total income* ***per year****, i.e., the sum of the incomes from the two or three harvests*.

………… dong per công

3.3. What is the amount of the rent that farmers pay to use your paddies?

Paddies with two rice cycles:………… dong per công

Paddies with two rice cycles:………… dong per công

3.4. Do you give this rent to the so-called “charities”?

☐Yes, always

☐Yes, sometimes

☐No

3.5. How often do you rent your paddies to other farmers?

☐ After each harvest

☐ After most harvests

☐ After some harvests

☐ Rarely

3.6. How often do different duck flocks have contacts on your rice paddies?

☐ Very often

☐ Sometimes

☐ Never

☐ I don’t know

3.7. How often does a duck flock use a paddy that has already been used by another flock during the same production cycle?

☐ Very often

☐ Sometimes

☐ Never

☐ I don’t know
