# Supplementary material for: Movement and contact patterns of long-distance free-grazing ducks and avian influenza persistence in Vietnam
Source: PLoS One. 2017 Jun 20;12(6):e0178241. doi: 10.1371/journal.pone.0178241 (PMC5478089; doi:10.1371/journal.pone.0178241)
Supplement: S3 File — (PDF) [file pone.0178241.s003.pdf]

Questionnaire for individual interviews with transporters of free grazing ducks

Location where the interview is conducted:
District: ……………………
Commune: ……………..

Sentences in italics are instructions for the interviewer.

1. Identification and characteristics of the household

1.1. What are your name and surname?
……………………………………………………………………………..
1.2. Where do you live? *This refers to the permanent home of the respondent*.
Province: …………………
District: ……………………
Commune: ……………..
Village: …..………………
1.3. How old are you? *Record the age of the respondent in years*.
………
1.4. Which ethnic group do you belong to?
☐ Kinh
☐ Khmer
☐ Mường
☐ Hoa
☐ Chăm
☐ Other…...
1.5. How many people belong to your household?
…………
1.6. *Ask the respondent to rank by income the productions and activities in which his/her household is involved. Ask him/her to think about the income over the last 12 months and record the rank number in the corresponding boxes*.
☐Rice
☐Other crops
☐Chicken
☐Ducks (common ducks or Muscovy ducks)
☐Geese
☐Pigs
☐Fish
☐Duck transport
☐Other: ………………………..
1.7. How important is the transport activity in term of income for your household?
☐ Very important
☐ Important
☐ Not important

For the remainder of the questions, ask the respondent to refer only to transport of free-grazing ducks between rice paddies. Some of the transporters might be involved in other activities, such as transporting ducks to markets, slaughter points, etc. (see last question).

2. Characteristics of the duck transportation

2.1. How many vehicles used for duck transport do you own?

……….. trucks

……….. boats

2.2. How many days do you work per month during the harvest season?

……..

2.3. How many days do you work per month outside the harvest season?

……..

2.4. How many journeys with duck flocks do you make in a typical working day during the harvest season?

………

2.5. How many journeys with duck flocks do you make in a typical working day outside the harvest season?

………

2.6. What is the maximum capacity of your vehicle(s)? *Record the maximum loading capacity in number of ducks*.

Vehicle 1: …………….

Vehicle 2: …………….

Vehicle 3: …………….

2.7. Out of 100 journeys, how many times do you transport flocks belonging to more than one farmer in the same vehicle?

……………..

3. Cleaning and disinfection of the vehicle

3.1. How often do you clean your vehicle?

………………………..

3.2. Do you disinfect your vehicle?

☐ Yes

☐ No

3.3. [Only if 3.2 is "Yes"] How often do you disinfect your vehicle?

…………………

3.4. [Only if 3.2 is "Yes"] Which disinfectant do you use?

…………………

3.5. Do you use your vehicle for other purposes than transporting free-grazing duck flocks?

☐ No

☐ Yes. *Record these other purposes:* ………………………………………………………………..
